# Effect of methylation of adenine N^6^ on kink turn structure depends on location

**DOI:** 10.1080/15476286.2019.1630797

**Published:** 2019-06-24

**Authors:** Saira Ashraf, Lin Huang, David M. J. Lilley

**Affiliations:** Cancer Research UK Nucleic Acid Structure Research Group, MSI/WTB Complex, The University of Dundee, Dundee, U.K

**Keywords:** RNA structure, RNA methylation, epigenetic modification, N^6^-methyladenine, kink-turn, X-ray crystallography

## Abstract

N^6^-methyladenine is the most common covalent modification in cellular RNA species, with demonstrated functional consequences. At the molecular level this methylation could alter local RNA structure, and/or modulate the binding of specific proteins. We have previously shown that *trans*-Hoogsteen-sugar (sheared) A:G base pairs can be completely disrupted by methylation, and that this occurs in a sub-set ofD/D k-turn structures. In this work we have investigated to what extent sequence context affects the severity with which inclusion of N^6^-methyladenine into different A:G base pairs of a standard k-turn affects RNA folding and L7Ae protein binding. We find that local sequence has a major influence, ranging from complete absence of folding and protein binding to a relatively mild effect. We have determined the crystal structure of one of these species both free and protein-bound, showing the environment of the methyl group and the way the modification is accommodated into the k-turn structure.

## Introduction

**Figure 1.**
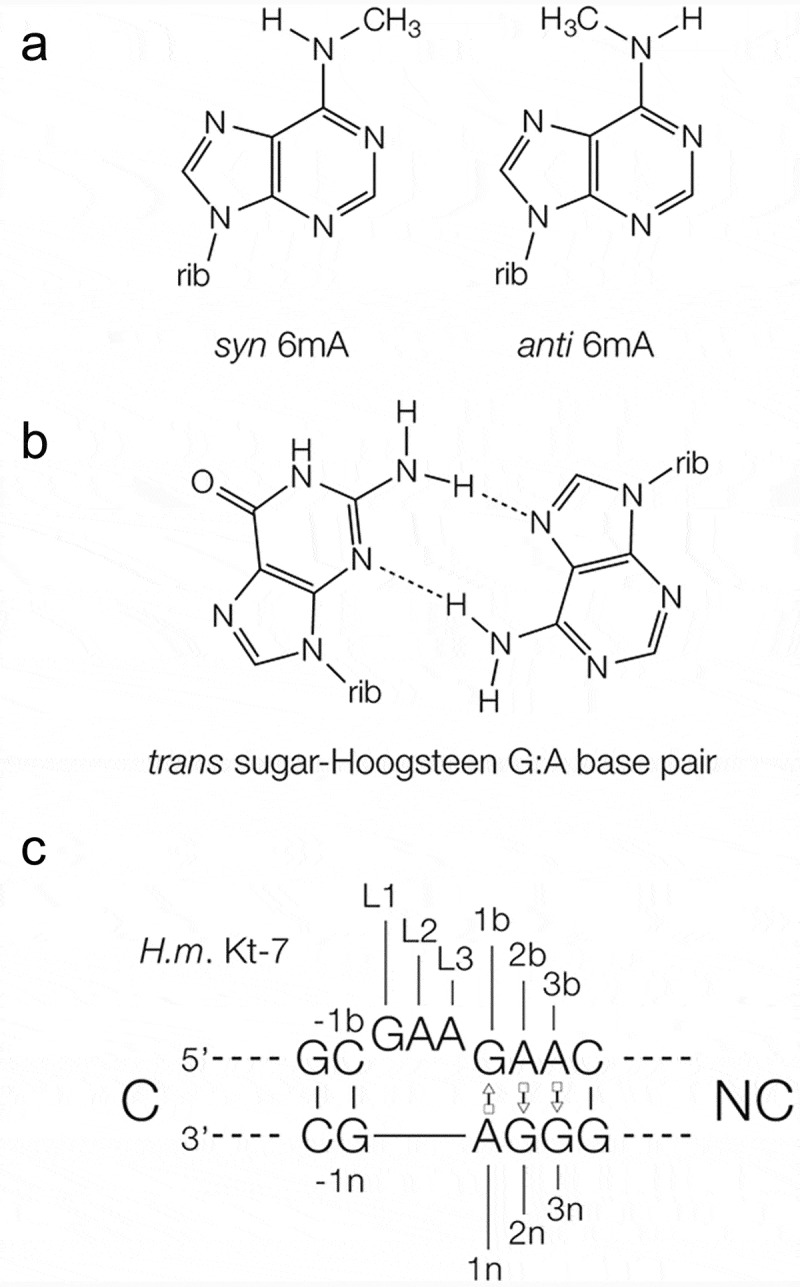
Methylation of adenine nucleobases, and the structure of kink turns in RNA. **(a)**. The chemical structure of the *syn* and *anti* conformers of N^6^-methyladenine. **(b)**. The structure of a *trans* sugar-Hoogsteen (sheared) G:A base pair. **(c)**. The sequence of *H. marismortui* Kt-7 k-turn, showing the nomenclature of the nucleotide positions and helices.

N^6^-methyladenine (Figure 1(a)) is very widespread in cellular RNA [1–3]. It is commonly found in eukaryotic mRNA, lncRNA species including Xist [2,3] and MALAT1 [4,5] as well as in viral RNA [6]. The level of modification is regulated by methyl transferase [7] and demethylase [8,9] enzymes. N^6^-methyladenine plays a role in the modulation of RNA stability [10], in gene regulation [11] and the control of translation efficiency found to be located in regions of mRNA suggestive of control functions [3].

In principle the presence of N^6^-methyladenine in cellular RNA could be recognized in two alternative ways. One is by the binding of proteins that are selective for the presence of the modified nucleobase (these are sometimes called ‘reader’ proteins), exemplified by the YTHDF2 protein [10,12,13] that has >3000 cellular targets. A second possibility is that the addition of the methyl group might directly affect the structure of the RNA and so alter its ability to assemble into a functional species [14–18]. We have previously used crystallography to show that N^6^-methyladenine is tolerated in *cis*-Watson-Crick A-U or A:G base pairs, but its inclusion can disrupt the formation of *trans*-sugar-Hoogsteen G:A base pairs (often called sheared base pairs; Figure 1(b)) [15]. If RNA structure was involved in the response to N^6^-methylation of the adenine, then sheared G:A base pairs would be the probable target for maximum effect. Sheared G:A base pairs are frequently found in key helical junctions in RNA structure, and most notably within the core of kink turns (k-turns) as tandem G:A, A:G base pairs [19,20].

k-turns are highly abundant structural motifs in RNA that generate a tight kink in the axis of RNA, mediating tertiary contacts and serving as protein binding sites [21]. k-turns play an important structural role in a number of key cellular RNA-protein complexes, including the box C/D snoRNP, where they bind the L7Ae (in archaea) or 15.5k protein (in eukaryotes) as the first stage of assembly. We showed that 15.5k binding and RNA folding of box C/D snoRNA k-turns is disrupted by N^6^-methylation of the adenine A1n (the nomenclature of nucleotide positions in the k-turn [22] is shown in Figure 1). Furthermore, using bioinformatics we found that a subset ofD/D snoRNA sequences have the sequence that would be required to make them a target for the METTL3-METTL14 methyltransferase, and about half of these are known to be methylated in the cell [21]. We also found that some tandem G:A, A:G sequences within Alu elements, including human SRP are subject to N^6^-methylation of adenine.

Thus we know that inclusion of N^6^-methyladenine in *tran*s-sugar-Hoogsteen G•A base pairs can have major structural consequences, and this could be the basis of their recognition and biological effects that follow from this. We now wish to know to what extent such effects might depend upon the local sequence context. We showed that N^6^-methylation of one critical adenine in the box C/D k-turns can prevent folding [21], and so is the basis of a probable regulatory mechanism in the cell. But what are the structural consequences of methylation of other adenine nucleobases ? We have therefore taken the well-characterised k-turn Kt-7 from *Haloarcular* *marismortui* to investigate the effect of inclusion of N^6^-methyladenine at different positions on folding induced by addition of metal ions and binding of the L7Ae protein. Kt-7 contains a number of adenine nucleotides, including those in three important *tran*s-sugar-Hoogsteen G:A base pairs. We have systematically introduced N^6^-methyladenine at four positions in Kt-7, including the three sheared G:A base pairs, and analysed the structure, folding and protein binding of the modified k-turns using fluorescence resonance energy transfer and X-ray crystallography. We find that the consequences of methylation strongly depend upon the location of the modified adenine within the structure.

## Results

### Systematic investigation of the effect of N^6^-methyladenine inclusion in a standard k-turn on folding and protein binding as a function of position

**Figure 2.**
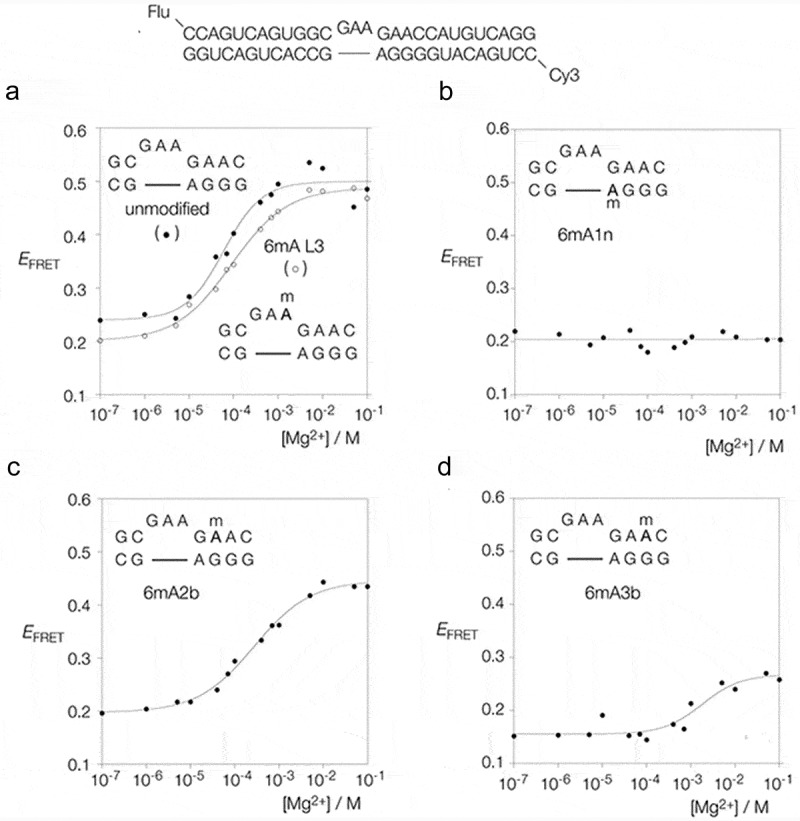
Analysis of the folding of Kt-7 induced by addition of Mg^2+^ ions, analysed by steady-state FRET. The sequence of the unmodified RNA is shown at the top; four variants in which a chosen adenine was replaced by N^6^-methyladenine were also prepared. Each species is labelled at the 5ʹ-termini by fluorescein (flu, donor) and Cy-3 (Cy3, acceptor) fluorophores. The efficiency of energy transfer (*E*_FRET_) is plotted as a functionofMg^2+^ concentration for each species. Where possible the data have been fitted to a two-state model of RNA folding Equation (1). **(a)**. Unmodified (filled circles) and L3-methylated (open circles) Kt-7. **(b)**. Kt-7 with A1n replaced by N^6^-methyladenine. This species failed to fold at any concentration of Mg^2+^. **(c)**. Kt-7 with A2b replaced by N^6^-methyladenine. **(d)**. Kt-7 with A3b replaced by N^6^-methyladenine.

We have synthesized a series of variants of the standard k-turn Kt-7 from *Haloarcula marismortui* in which adenine has been substituted by N^6^-methyladenine. Four positions have been investigated. These are the adenine nucleotides of the three sheared G:A base pairs (A1n, A2b and A3b) and that at the 3ʹ end of the loop (L3) (Figure 1(c) shows the nomenclature of the nucleotide positions [22]). Folding has been investigated using fluorescence resonance energy transfer (FRET), and protein binding by retardation of electrophoretic mobility in polyacrylamide. Folding of Kt-7 has been studied using a 25 bp RNA duplex with a central Kt-7 sequence, with fluorescein (donor) and Cy-3 (acceptor) fluorophores attached to the 5ʹ-termini (Figure 2). When the k-turn adopts the kinked conformation the distance between the fluorophores shortens so that the efficiency of energy transfer rises. FRET efficiency (*E*_FRET_) has been measured by steady-state fluorimetry using the acceptor-normalization method [23].

### Effect of N^6^-methyladenine inclusion at various positions in Kt-7 on ion-induced folding

Figure 2(a) shows a plot of *E*_FRET_ as a function of Mg^2+^ ion concentration for unmodified Kt-7 (filled circles). At low concentrations of the ion the population is predominantly unfolded and *E*_FRET_ is below 0.25. This rises to 0.5 as the k-turns in the population fold in response to increased Mg^2+^ ion concentration. The data fit a two-state folding model, from which a [Mg^2+^]_1/2_ = 68 µM is calculated, in good agreement with earlier measurements [22]. Inclusion of N^6^-methyladenine at the L3 position of the loop resulted in comparable folding, with a very similar change in *E*_FRET_. Fitting the data gives [Mg^2+^]_1/2_ = 90 µM, i.e. folding requires a slightly higher concentration of the ion for this modification.

Inclusion of N^6^-methyladenine at the A1n position resulted in no folding being observed up to a concentration of 0.1 M Mg^2+^ (Figure 2(b)). This modification totally prevented the folding of Kt-7 in response to the addition of metal ions.

By contrast, when N^6^-methyladenine was substituted at the A2b position the resulting Kt-7 variant underwent Mg^2+^-induced folding (Figure 2(c)). Compared to unmodified Kt-7 the maximum *E*_FRET_ value was slightly lower at 0.43, and the [Mg^2+^]_1/2_ = 275 µM, a four-fold higher concentration than required by unmodified Kt-7.

Lastly inclusion of N^6^-methyladenine at the A3b position had a marked effect on Mg^2+^-induced folding (Figure 2(d)). Only a small change in *E*_FRET_ was observed, and this required a very high concentration of Mg^2+^ ions, corresponding to [Mg^2+^]_1/2_ = 1.8 mM.

The titrations have been repeated a total of three times, and the data are completely reproducible. We note that all Kt-7 constructs exhibit an *E*_FRET_ of 0.2 in the absence of Mg^2+^ ions. This is consistent with a duplex that is bent by the presence of a 3-nucleotide bulge, but not kinked in the k-turn conformation. The structure in the absence of divalent ions is unaffected by N^6^-methyladenine inclusion at any position.

In summary, the effect of N^6^-methylation of adenine on the ion-induced folding of Kt-7 depends strongly on the position of the modified nucleotide. While the effect of adenine modification in the L3 position is minimal, that in the three G:A base pairs is strikingly different. Modification of A1n totally prevents folding, A2b undergoes some ion-induced folding and A3b only exhibits very weak folding. Thus the order of severity of folding impairment is A1n > A3b > A2b.

### Effect of N^6^-methyladenine inclusion at various positions in Kt-7 on L7Ae binding and induced folding

**Figure 3.**
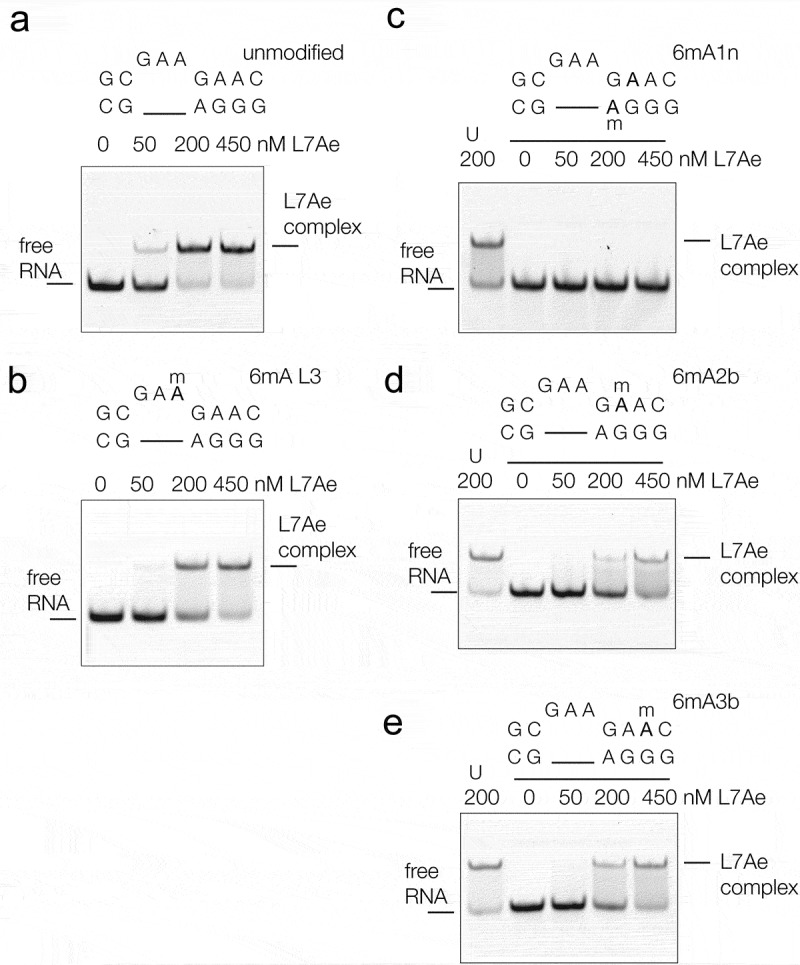
The binding of *A. fulgidus* L7Ae to Kt-7 analysed by electrophoresis in polyacrylamide gels. 200 nM Cy3-labeled RNA was incubated with the indicated concentrations of *Af*L7Ae in the range 0–450 nM before being electrophoresed in a 10% native polyacrylamide gel. Fluorescent RNA was visualized by fluorimaging. a. Unmodified Kt-7 incubated with L7Ae. **b**–**e**. Kt-7 modified by inclusion of N^6^-methyladenine at the L3, 1n, 2b and 3b positions respectively. In the gels shown in c-e the left-most track (U) contains the result of incubating unmodified Kt-7 with 200 nM L7Ae.

**Figure 4.**
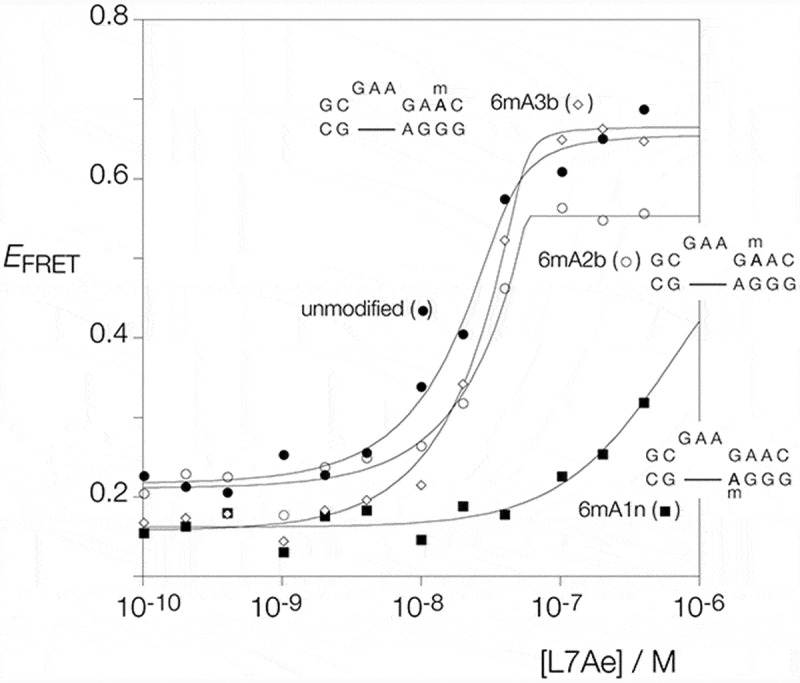
Analysis of the folding of Kt-7 induced by the binding of *Af*L7Ae, analysed by steady-state FRET. *Af*L7Ae protein was titrated in to fluorescein-Cy-3-labeled RNA (see Figure 2) and *E*_FRET_ calculated. The experiment was repeated using unmodified Kt-7 (filled circles), and Kt-7 with inclusion of N^6^-methyladenine at the 1n (filled squares), 2b (open circles) and 3b positions (open diamonds). *E*_FRET_ is plotted as a function of *Af*L7Ae concentration, and the data have been fitted to Equation (2).

We next examined the ability of the various N^6^-methyladenine-modified Kt-7 variants to bind L7Ae from *Archeoglobus fulgidus* (*Af*L7Ae), and undergo folding in response to binding. *Af*L7Ae binding was studied using gel electrophoresis (Figure 3) and *Af*L7Ae-induced folding was studied by FRET (Figure 4). Both experiments were performed using the same RNA fluorescent constructs as those used to analyse ion-induced folding.

Fluorescent RNA was incubated with *Af*L7Ae, applied to a 10% polyacrylamide gel and electrophoresed under non-denaturing conditions (Figure 3). Incubation with unmodified Kt-7 results in the observation of a discrete retarded complex under stoichiometric conditions (Figure 3(a)). We know from previous measurements of binding and dissociation rates that *Af*L7Ae binds to Kt-7 with sub-pM affinity [24], and thus under the conditions of these experiments binding is stoichiometric. Addition of *Af*L7Ae also led to folding of Kt-7, reaching an *E*_FRET_ = 0.66 (Figure 4), showing that binding leads to the folding of Kt-7 as shown previously [20,25]. The *Af*L7Ae binding characteristics of Kt-7 containing N^6^-methyladenine at the L3 position was closely similar to that of the unmodified k-turn (Figure 3(b)).

Turning to the adenine nucleotides of the three sheared G:A base pairs we find that the effect of N^6^-methylation on *Af*L7Ae binding depends upon position. Upon inclusion of N^6^-methyladenine at the A1n position no complex formation could be detected (Figure 3(c)). A small increase in *E*_FRET_ was observed at concentrations of *Af*L7Ae in excess of 100 nM (Figure 4), but these data do not have the characteristics of k-turn formation. By contrast some discrete complex formation was observed on incubation of *Af*L7Ae with Kt-7 containing N^6^-methyladenine at the A2b (Figure 3(d)) and A3b positions (Figure 3(e)). Both require higher concentrations of *Af*L7Ae, consistent with lowered affinity. The A2b and A3b-modified Kt-7 species both undergo folding in response to protein addition, achieving *E*_FRET_ = 0.55 (A2b) and 0.67 (A3b) (Figure 4). A greater relative extent of folding is achieved for these variants on addition of L7Ae, compared to addition of Mg^2+^ ions. This will be a result of the high affinity of L7Ae for Kt-7 [24], with the free energy of L7Ae binding providing a substantial driving force for folding.

### The crystal structure of Kt-7 with N^6^-methyladenine at the 2b position

**Figure 5.**
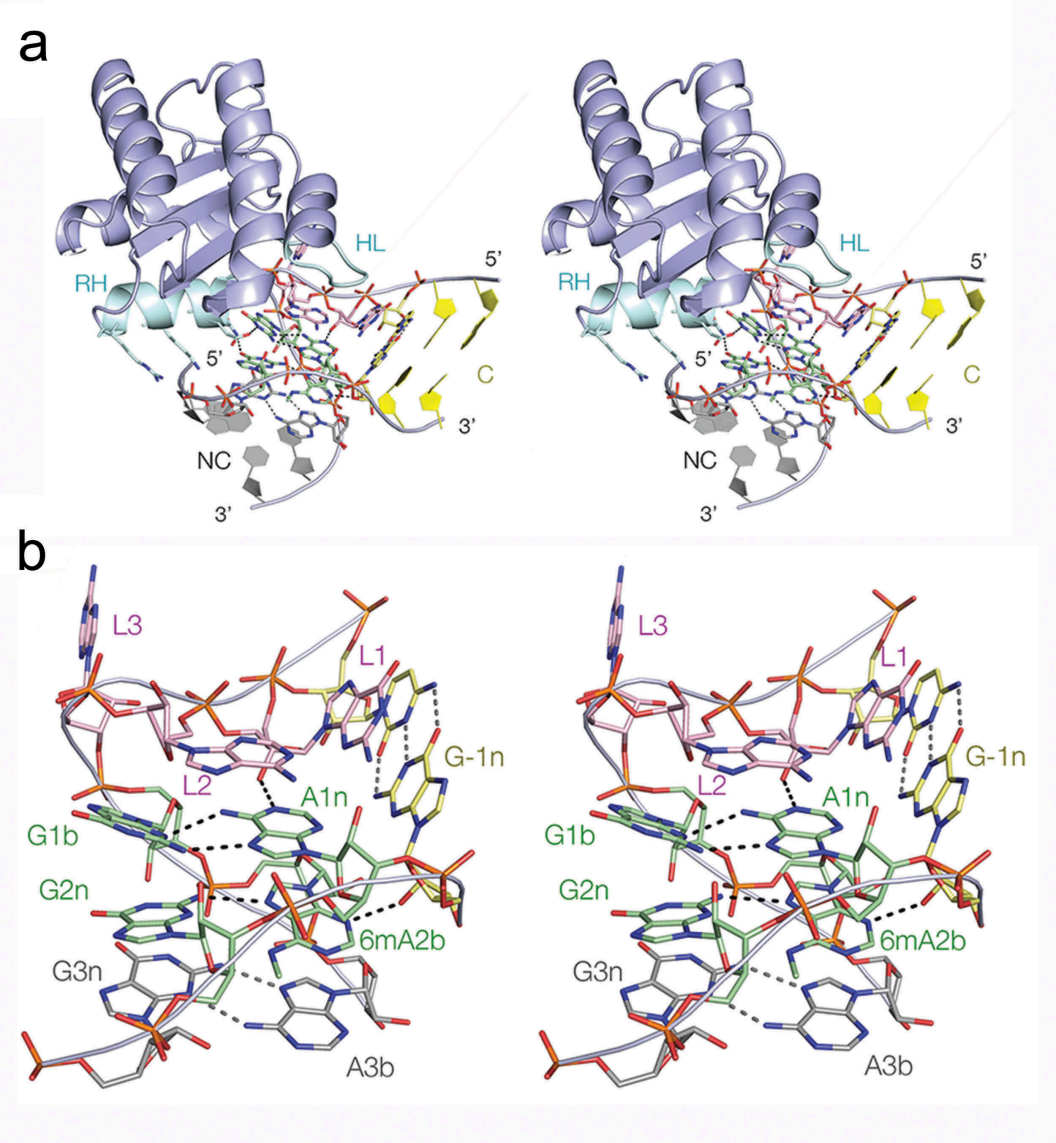
The crystal structure of Kt-7 with N^6^-methyladenine at the 2b position bound to *Af*L7Ae. Parallel-eye stereoscopic views are shown. **(a)**. Overall view of the structure seen from the side of the non-bulged strand. The recognition helix (RH) and hydrophobic loop (HL) of the protein are highlighted in cyan. Side chains of the recognition helix that interact with the RNA are shown in stick form. **(b)**. The structure of the core of the k-turn in the complex. The RNA adopts a standard N3-conformation k-turn structure.

Given that the N^6^-methyladenine 2b Kt-7 underwent folding on addition of Mg^2+^ ions, we investigated the structure using X-ray crystallography. Crystals of self-complementary RNA containing two Kt-7 k-turns were obtained with and without bound *Af*L7Ae, at resolutions of 1.99 and 2.09 Å respectively. The structure of the k-turn containing N^6^-methyladenine 2b is closely similar in the L7Ae bound and protein free states, superimposing with an all-atom RMSD of 0.52 Å (Figure S1). The structure of the complex is shown in Figure 5. The overall structure is very similar to that of *Af*L7Ae bound to unmodified Kt-7 [26]. The protein places its recognition helix in the widened major groove on the outside of the k-turn, making specific interactions with the conserved guanine nucleobases of the G:A base pairs and further non-specific backbone contacts (Figure 5(a)). Its hydrophobic loop is located over the L1 and L2 nucleobases. The overall shape of the k-turn is closely similar to that of unmodified Kt-7 (Figure S2A), and the structure of the core is standard (Figure 5(b)). The key cross-strand hydrogen bonds from L1 O2ʹ to A1n N1 and from -1n O2ʹ to A2b N3 are present; thus the structure adopts its normal N3 conformation [27]. The overall conformation of Kt-7 has accommodated the N^6^-methyladenine at the 2b position.

**Figure 6.**
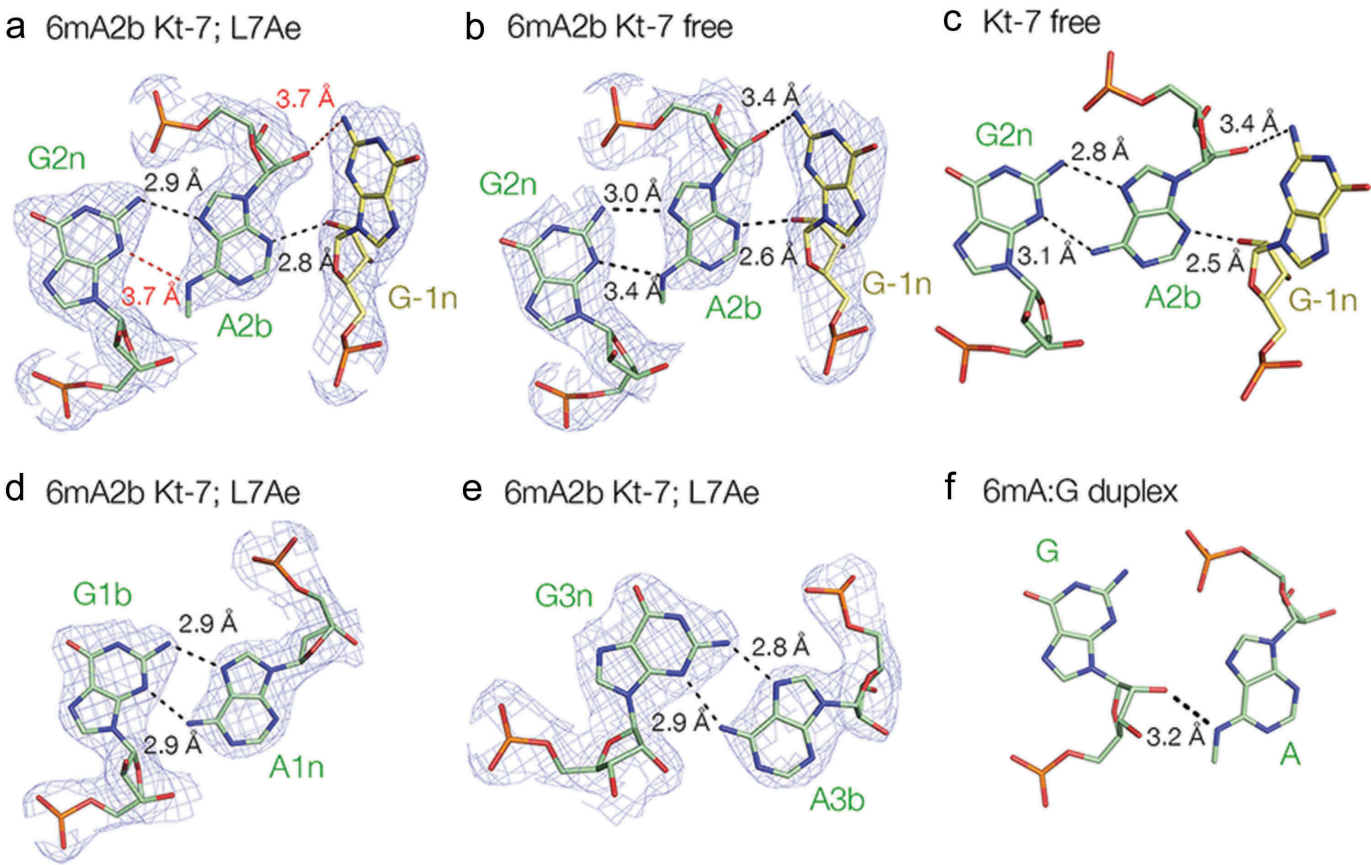
A2b:G2n base pairs found in different structures and positions. For those taken from the current study, the 2**F**_o_-**F**_c_ electron density map contoured at 1.2 σ is shown. **(a)**. The 2b:2n.-1n interaction in the structure of the complex of A2n N^6^-methyladenine Kt-7 with *Af*L7Ae. Potential hydrogen bonds are shown as broken lines, with distances > 3.3 Å drawn red. **(b)**. The 2b:2n.-1n interaction in the structure of protein-free A2n N^6^-methyladenine Kt-7. **(c)**. The 2b:2n.-1n interaction in the structure of protein-free unmodified Kt-7 (PDB 4CS1) [39]. **(d)**. The 1b:1n interaction in the structure of the complex of A2n N^6^-methyladenine Kt-7 with *Af*L7Ae. **(e)**. The 3b:3n interaction in the structure of the complex of A2n N^6^-methyladenine Kt-7 with *Af*L7Ae. **(f)**. The G:A interaction in an RNA duplex containing tandem G:A, A:G base pairs flanked by G:U base pairs (PDB 5LR3) [15]. Note that in this structure there is no hydrogen bonding between the two nucleobases.

However, close inspection of the A2b:G2n base pair reveals some local conformational readjustment (Figure 6). The A2b:G2n base pair in the protein-bound (Figure 6(a)) and free Kt-7 (Figure 6(b)) structures can be compared with those of an unmodified Kt-7 A2b:G2n base pair (Figure 6(c); PDB 4CS1), as well as the 1b:1n and 3b:3n base pairs in the N^6^-methyladenine 2b Kt-7 bound to *Af*L7Ae (Figure 6(d and e) respectively). In the 2b:2n base pair of the unmodified Kt-7, as well as the 1b:1n and 3b:3n base pairs of the modified Kt-7 both hydrogen bond lengths fall in the normal range of 2.8–3.1 Å. However that of N^6^-methyladenine 2b Kt-7 bound to *Af*L7Ae it is 3.7 Å and is thus beyond the range normally considered to be stably bonded. The distance between G-1n N2 and A2b O2ʹ is also 3.7 Å. Superposition of the structures of N^6^-methyladenine 2b Kt-7 and unmodified Kt-7 (Figure S2B) shows that the disruption of the hydrogen bonding is primarily due to an in-plane rotation of A2b, leaving G2n essentially unaltered. The resolution of the protein-free N^6^-methyladenine 2b Kt-7 is a little lower, and thus the position of the methyl group less well defined. Nevertheless an 8° rotation of the A2b nucleobase about the ribose and lengthening of the A2b N6 – G2n N3 distance is apparent (Figure 6(b)).

## Discussion

The most clear-cut conclusion emerging from this work is that the structural influence resulting from the inclusion of N^6^-methyladenine in an A:G base pair depends markedly on its position. We have previously shown that N^6^-methyladenine is readily accommodated in *cis*-Watson-Crick A-U and A:G base pairs, but that *trans*-Hoogsteen-sugar A:G base pairs can be strongly disrupted [15] (Figure 6(f)). In this work we now extend this, finding that the influence of N^6^-methyladenine on *trans*-Hoogsteen-sugar A:G base pairs varies greatly with structural context.

Comparing the three sheared A:G base pairs of Kt-7 we find that k-turn folding and L7Ae binding are disrupted to very different extents by inclusion of N^6^-methyladenine, covering almost the full range of possible disruption. Inclusion of N^6^-methyladenine at the 1n position completely prevents ion induced folding, binding of *Af*L7Ae and concomitant folding. Inclusion at the 2b and 3b positions had much smaller effects, both binding *Af*L7Ae and becoming folded. The main difference between these two modifications was found in ion-induced folding, where the 3b-modified Kt-7 folded much better than the 2b-modified form.

The strong effect of inclusion of N^6^-methyladenine at the 1n position in Kt-7 is similar to our previous observation that the equivalent change inD/D and box C′/D′ k-turns prevented human 15.5k protein (the mammalian homolog of L7Ae) from binding or folding the RNA [15]. Why then does inclusion of N^6^-methyladenine at the 1n position have such a major effect on folding compared to the 2b or 3b positions ? Methyl groups in the *syn* position of N6 at either A2b or A3b are located in the minor groove, projecting out into the solvent although relatively close to the backbone of the opposite strand (Figure S3A). In contrast, a methyl group in the equivalent position of A1n lies in a very constrained environment (Figure S3B) located only 2.2 Å from O4ʹ of G1b. This would be expected to be very disruptive for the k-turn structure, explaining our failure to observe folding. The difference between the effect of methylation at the A2b and A3b positions appears to be due to differences in ribose conformation on the opposing strand. The AN6 – O2ʹ distances are 5.5 and 3.0 Å for the 2b:2n and 3b:3n base pairs respectively.

In eukaryotes the METTL3-METTL14 methyl transferase complex methylates N6 of adenine located in the consensus sequence DRACH (where D denotes A, G or U, R is A or G and H is A, C or U), with GAC as the most common site of methylation [2,3]. Thus in order for A1n to be methylated, the -1n nucleotide should be C. While in many k-turns this is G, using bioinformatic analysis we showed previously that a sub-set of human box C/D sequences have C at the -1n position, and that approximately half of these have N^6^-methyladenine at the 1n position [15]. Since this prevents 15.5k protein binding and folding of the k-turn this immediately suggests a mechanism of methyl-dependent regulation of box C/D snoRNP assembly. This raises the question of whether or not N^6^-methyladenine could be incorporated at the 2b position of cellular k-turn motifs, that would require the presence of C at the 3b position. In human ribosomal RNA species Kt-23 and Kt-42 both have 3b = C sequences [28], and the RMBase data base [29] shows that both are methylated at the 2b position (Figure S4). It is therefore possible that N^6^-methyladenine 2b inclusion could play a role in ribosome assembly.

In summary, while inclusion of N^6^-methyladenine into *trans*-Hoogsteen-sugar A:G (sheared) base pairs can have major structural effects, the extent of the influence depends strongly on sequence context. Thus there is strong potential for N^6^-methylation of adenine to exert differential structural effects and thus varying sensitivity of the RNA to functional modulation by reversible methylation.

## Materials and methods

### RNA synthesis and deprotection

Oligonucleotides were synthesized using *t*-BDMS phosphoramidite chemistry [30] as described in Wilson et al. [31], implemented on an Applied Biosystems 394DNA/RNA synthesizer. RNA was synthesized using Pac-A-CE, Ac-C-CE, iPr-Pac-G-CE and U-CE ribonucleotide phosphoramidites with 2ʹO-*tert*-butyldimethyl-silyl (*t*-BDMS) protection [32,33] (Link Technologies). The N^6^-methyl-A-CE phosphoramidite with *t*-BDMS protection was obtained from Glen Research. Fluorescein (Link Technologies) and Cy3 (GE Healthcare) were attached to the 5′ termini of the oligonucleotides as phosphoramidites in the final cycle of the synthesis, as required.

Oligonucleotides were deprotected in 25% ethanol/ammonia solution for 3h at 20°C. Oligonucleotides containing N^6^-methyladenine were further deprotected for 2h at 65°C. After deprotection, the oligonucleotides were evaporated to dryness. They were re-dissolved in 115 μl DMSO (Sigma-Aldrich) to which was added 60 μl triethylamine (TEA) (Sigma-Aldrich) and 75 μl 1 M triethylamine trihydrofluoride (TEA, 3HF) (Sigma-Aldrich) and incubated at 65°C for 2.5 h to remove the *t*-BDMS protecting groups. Thereafter, the samples were cooled on ice for 10 min and 250 μl RNA quenching buffer (Glen Research) was added. The oligonucleotides were then desalted using NAP-10 columns (GE Healthcare).

### RNA purification and hybridisation

Oligonucleotides were purified by denaturing gel electrophoresis in 20% acrylamide: bis-acrylamide 19:1 (Scientific Laboratory Supplies). Electrophoresis was performed in 90 mM Tris.borate (pH 8.5) 10 mM EDTA (TBE), at 25 W for approximately 3 h. RNA was visualized by UV shadowing and bands corresponding to full length RNA product were excised and electroeluted into TBE at 150 V at 20°C. The RNA was then precipitated with ethanol. The fluorophore-labelled oligonucleotides were subjected to further purification by reversed-phase HPLC on a C18 column (ACE 10–300, Advanced Chromatography Technologies), using an acetonitrile gradient with an aqueous phase of 100 mM triethylammonium acetate (pH 7.0) (Fisher Scientific). Collected fractions were evaporated to dryness and re-suspended in 120 μl ultrapure water.

Duplex species were prepared by mixing equimolar quantities of the appropriate oligonucleotides in TBE, 25 mM NaCl, and annealed by slow cooling from 95°C to 4°C. Hybridised RNA was purified by native gel electrophoresis in 12% acrylamide:bis-acrylamide 29:1 (Scientific Laboratory Supplies), in TBE, 25 mM NaCl, with buffer recirculation. Electrophoresis was performed at 150V at 4°C for approximately 6 h. Bands corresponding to duplex RNA species were excised from the gel and electroeluted into 0.25X TBE buffer at 100 V at 4°C, followed by ethanol precipitation and air-drying at 4°C.

### L7Ae expression and purification

*A. fulgidus* L7Ae cloned into a modified pET-Duet1 plasmid (Novagen) [34] was expressed as a hexahistidine fusion protein in *Escherichia coli* BL21-Gold (DE3) pLysS cells (Stratagene), and purified as reported previously [26].

### FRET analysis of k-turn folding

FRET efficiency was measured from a series of RNA duplex species (Figure 2), terminally 5ʹ-labelled with fluorescein on the bulged strand and Cy3 on the non-bulged strand, containing central Kt-7 k-turn sequence and its methylated variants. The unmodified strands were (all sequences written 5ʹ to 3ʹ): Flu-CCAGUCAGUGGCGAAGAACCAUGUCAGG and Cy3-CCUGACAUGGGGAGCCACUGACUGG. Modified versions of this species were constructed as indicated in the text. Absorption spectra were measured by re-dissolving the ethanol-precipitated hybrid RNA in 120 μl 90 mM Tris-borate (pH 8.3) and measuring the absorbance in a 50 mm path length cuvette using a Thermo Scientific NanoDrop 2000c spectrophotometer. Spectra were de-convoluted using a corresponding RNA species labelled only with Cy3, and fluorophore absorption ratios calculated using a program implemented in MATLAB. Fluorescence spectra were recorded in 90 mM Tris-borate (pH 8.3) at 4°C using an SLM-Aminco 8100 fluorimeter. Spectra were corrected for lamp fluctuations and instrumental variations, and polarization artifacts were avoided by setting excitation and emission polarizers crossed at 54.7°. Values of FRET efficiency (*E*_FRET_) were measured using the acceptor normalization method [23] implemented in MATLAB. *E*_FRET_ as a function of Mg^2+^ ion concentration was analysed on the basis of a model in which the fraction of folded molecules corresponds to a simple two-state model for ion-induced folding, i.e.

(1) $$E_{FRET}=E_{0}+\Delta E_{FRET}.K_{A}{\left[Mg^{2+}\right]}^{n}/\left(1\;+K_{A}{\left[Mg^{2+}\right]}^{n}\right)$$

where *E_0_* is the FRET efficiency of the RNA in the absence of added metal ions, *ΔE*_FRET_ is the increase in FRET efficiency at saturating metal ion concentration, [Mg^2+^] is the prevailing Mg^2+^ ion concentration, *K_A_* is the apparent association constant for metal ion binding and *n* is a Hill coefficient. Data were fitted to this equation by nonlinear regression. The metal ion concentration at which the transition is half complete is given by [Mg^2+^]_1/2_ = (1/*K_A_*)^1/*n*^.

The same hybrid RNA species were used to observe L7Ae binding-induced folding, and FRET efficiency was calculated using the same approach. *Af*L7Ae protein was titrated in to the RNA and added to a final concentration of 2 µM. *E*_FRET_ as a function of *Af*L7Ae concentration was fitted to:

(2) $$\begin{array}{rl} & E_{FRET}=E_{0}\\ & +\Delta E_{FRET}\frac{\left(1+K_{A}P_{T}+K_{A}R_{T}\right)-\sqrt{{\left(1+K_{A}P_{T}+K_{A}R_{T}\right)}^{2}-4R_{T}K_{A}^{2}P_{t}}}{2R_{T}K_{A}} \end{array}$$

where *E_0_* is the initial FRET efficiency in the absence of added protein, *∆E_FRET_* is the full range of the change in FRET efficiency, *K_A_* is the apparent association constant and *P_T_* and *R_T_* are the total concentration of *Af*L7Ae and RNA respectively.

### Electrophoretic analysis of protein binding to k-turn

Binding of *Af*L7Ae protein to the Kt-7 k-turn RNA and its modified versions was analysed by native gel electrophoresis. 200 nM Cy3-labeled RNA (the same species as used in the FRET experiments) was incubated in 45 mM Tris-borate (pH 8.3) with various concentrations of the *Af*L7Ae protein in a final volume of 10 µl at 7°C for at least 1 h. An equal volume of loading buffer containing 10% glycerol and 45 mM Tris-borate (pH 8.3) was added, and the samples were electrophoresed in 10% native polyacrylamide gels in 45 mM Tris-borate (pH 8.3) at 20 V/cm at 7°C for 1 h. Fluorescent RNA was visualized using a Typhoon 9500 fluorimager (GE Healthcare).

### Crystallization, structure determination, and refinement

A 19 nt RNA of sequence GGCGAAG(6mA)ACCGGGGAGCC was crystalized (6mA = N^6^-methyladenine). This sequence is self-complementary and forms two Kt-7 k-turns with two-fold symmetry. A solution of 1 mM RNA in 5 mM Tris.HCl (pH 8.0), 100 mM NaCl, 10 mM MgCl_2_ was incubated for 1 min at 95°C. Crystals were grown by vapor diffusion using drops prepared by mixing 1 μL of the RNA with 1 μL of a reservoir solution comprising 0.1 M KCl, 0.002 M spermine tetrahydrochloride, 0.05 M Bis-Tris (pH 8.0) and 15% w/v PEG 2000 MME at 17°C. Crystals appeared after 10 days. They were transferred into the reservoir solution with 30% PEG200 for ~ 3 s. The crystals were flash frozen by mounting in nylon loops and plunging into liquid nitrogen.

A self-complementary 21 nt RNA oligonucleotide CGGCGAAG(6mA)ACCGGGGAGCCG was crystalized as a complex bound to *Af*L7Ae. This RNA has additional C-G base pairs at both ends. A solution of 0.5 mM RNA and 0.5 mM *Af*L7Ae in 5 mM Tris.HCl (pH 8.0), 100 mM NaCl, 10 mM MgCl_2_ was incubated for 1 min at 85°C followed by slow cooling. Crystals were grown by vapor diffusion using drops prepared by mixing 1.0 μL of the RNA–protein complex with 1 μL of a reservoir solution comprising 0.01 M spermine tetrahydrochloride, 0.05 M MES (pH 6.5), 25% v/v PEG 400 at 17°C. Crystals appeared after 5 days.

All the crystals were characterized in-house with a MicroMax HF007 copper rotating anode X-ray generator equipped with an ACTOR sample changer system and a Saturn 944HG+ CCD detector (Rigaku). Suitable crystals were stored and subsequently used to measure full datasets on beamline I03 of Diamond Light Source (Harwell, UK. Data were processed by XIA2 [35].

The structures of the HmKt-7 variant A2bm6A without and with *Af*L7Ae protein were determined by molecular replacement with Phenix Phaser. The k-turn search model was taken from PDB 4CS1, and the k-turn *Af*L7Ae complex model was taken from PDB 4BW0. The resulting electron density maps revealed the remaining RNA density, and the model was built de novo on the basis of the difference map. Structural models were built in Coot [36] and refined by Phenix [37]. Omit maps were calculated using Phenix. Model geometry and the fit to electron-density maps were monitored with MOLPROBITY [38] and the validation tools in COOT.

## Data Availability

Atomic coordinates and structure factor amplitudes have been deposited with the PDB with accession code 6Q8U and 6Q8V.
