# Supplementary material for: Effect of methylation of adenine N6 on kink turn structure depends on location
Source: RNA Biol. 2019 Jun 24;16(10):1377–85. doi: 10.1080/15476286.2019.1630797 (PMC6779385; doi:10.1080/15476286.2019.1630797)
Supplement: Supplemental Material [file krnb-16-10-1630797-s001.pdf]

Effect of methylation of adenine N6 on kink turn structure depends on location

S. Ashraf, L. Huang and D. M. J. Lilley

SUPPLEMENTARY INFORMATION

SUPPLEMENTARY FIGURES

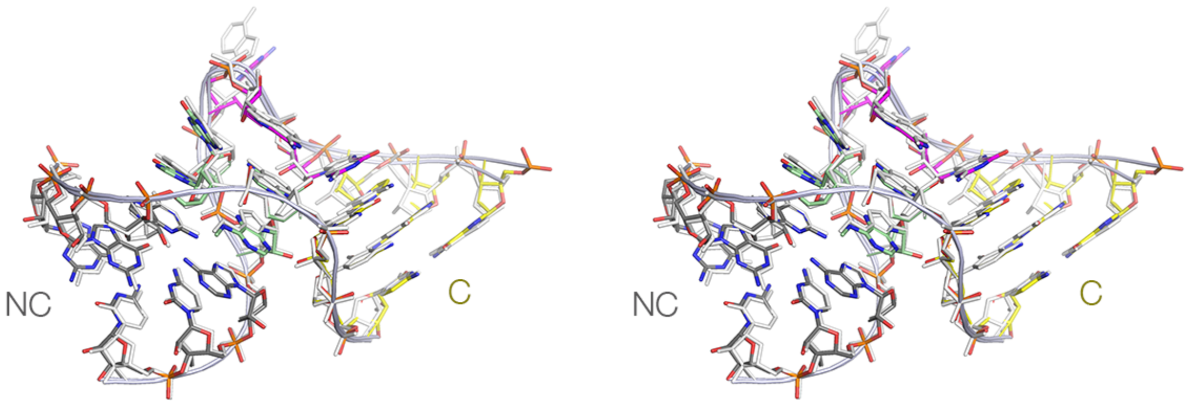

Figure S1. Superposition of the structures of Kt-7 with N^6-methyladenine at the 2b position as free RNA and bound to L7Ae. Parallel-eye stereoscopic view of the overall k-turn structures viewed from the side of the unbulged strand is shown. The L7Ae-bound structure (PDB ID 6Q8U) is shown in our standard k-turn coloring, while the free RNA (PDB ID 6Q8V) is colored grey. The free and bound structures are well superimposed, with an all-atom RMSD = 0.52 Å.

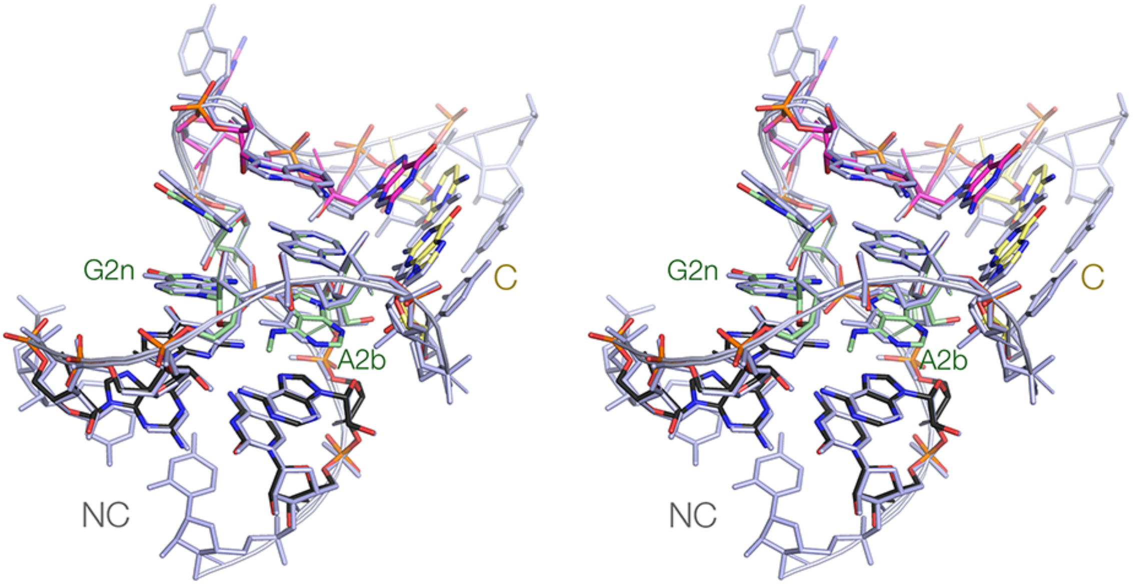

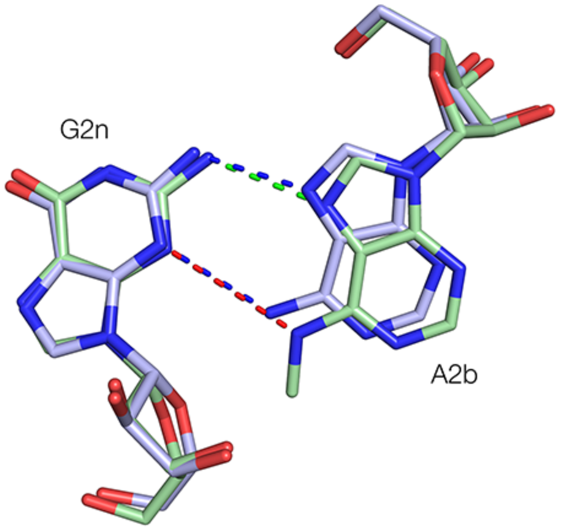

Figure S2. Superposition of the structures of Kt-7 with and without inclusion of N^6-methyladenine at the 2b position. A parallel-eye stereoscopic view of the overall k-turn structures views from the side of the unbulged strand is shown at the top. The methylated structure is shown using our conventional coloring while the unmodified structure is drawn grey. The superimposed A2b:G2n pairs are shown below. Note that while the guanine nucleotides are well superimposed, the adenines have diverged. N^6-methyladenine 2b has rotated 6° anticlockwise, so lengthening the A2b N6 to G2n N3 distance to 3.7 Å (shown red).

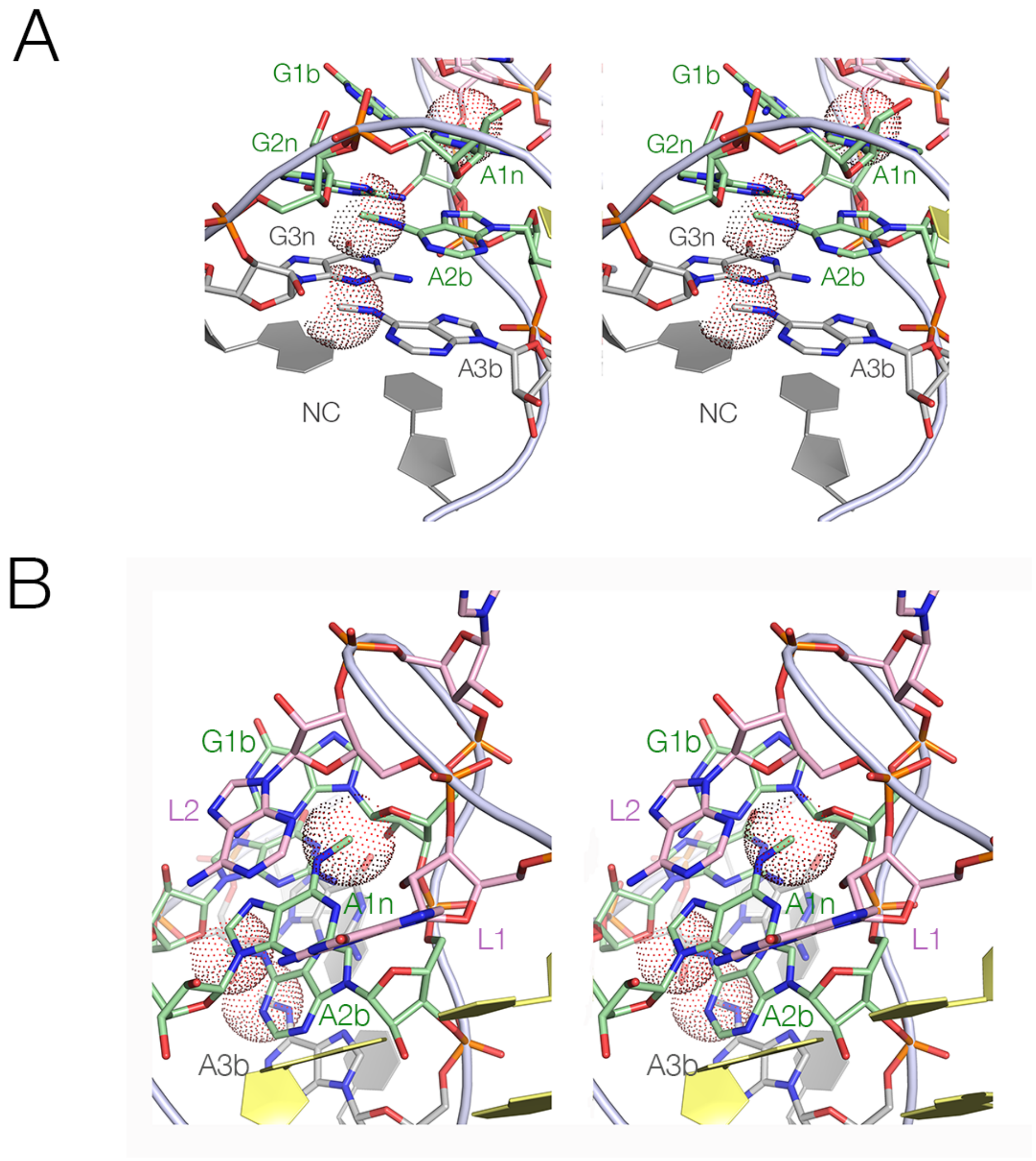

Figure S3. The steric environment of methyl groups attached to N6 of A1n, A2b and A3b in Kt-7. Methyl groups were modeled on to the *syn* positions of N6 of A1n, A2b and A3b in a high resolution structure of Kt-7 (PDB 4CS1). Parallel-eye stereo views of the resulting structure, with the volumes of the methyl group indicated by the dots.

A. View into the minor groove of the C helix, where the position of the methyl groups on A2b and A3b can be seen unimpeded.

B. View down onto the loop of the k-turn, showing the cramped environment of the methyl group attached to A1n. Note that it is particularly close to the ribose ring of G1b.

A

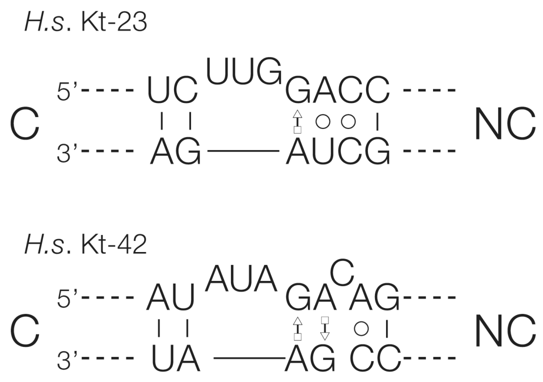

B

Kt	gene	Mod ID	Xsome	position	Support no	Sequence (6mA)
*Hs*Kt-23	SSU-rRNA_Hsa	6mA_site_48600	chr16	33963467	9	GCTAGAGGTGAAATTCTTGGACCGGCGCAGTTTGGACCAGA
*Hs*Kt-42	LSU-rRNA_Hsa	6mA_site_66141	chr19	24184402	5	AAATGTGTTGGTTGATATAGACAGCAGGACGGTGGGCATGG
*Hs*Kt-42	LSU-rRNA_Hsa	6mA_site_66488	chr19	36066543	15	AAAGGTGTTGGTTGATATAGACAGCAGGACGGTGGCCATGG
*Hs*Kt-42	LSU-rRNA_Hsa	6mA_site_75615	chr2	133039022	12	AAAGGTGTTGGTTGATATAGACAGCAGGATGGTGGCCATGG
*Mm*Kt-23	SSU-rRNA_Hsa	6mA_site_190217	chr17	39847330	3	GCTAGAGGTGAAATTCTTGGACCGGCGCAAGACGGACCAGA

Figure S4. K-turns of the human ribosome that undergo N^6-methylation at A2b.

A. Sequences of Kt-23 and Kt-42 drawn in their k-turn secondary structure. In both sequences G1bA2b are followed by C, so creating a GAC prefered target for the METTL3-METTL14 methyltransferase.

B. Bioinformatic analysis of methylation, extracted from the RMBase data base. This shows there are multiple observations of N^6-methyladenine occurrence, at the sequences highlighted in the final column (shown as DNA sequence). The putative k-turn region is highlighted green, and the N^6-methyladenine written red. *Mm*Kt-23 is the corresponding sequence found in the mouse (*Mus musculus*).

.

SUPPLEMENTARY TABLES

PDB ID	protein	mother liquor	resolution	space group	beamline
6Q8U	*Af*L7Ae	10 mM spermine HCl<br>50 mM MES (pH 6.5)<br>25 % v/v PEG 400	1.99 Å	$P2_12_12_1$	Diamond I03
6Q8V	-	0.1 M KCl<br>2 mM spermine HCl<br>50 mM Tris-Bis (pH 7.0)<br>15 % w/v PEG 2000 MME	2.09 Å	$P6_322$	Diamond I03

Table S1. Summary of the RNA species, ligands and crystallization conditions used in these experiments, and the crystals obtained.

PDB	6Q8U	6Q8V
	HmKt-7 variant A2b 6mA bound with *Af*L7Ae protein	HmKt-7 variant A2b 6mA
**Data collection**		
Space group	$P2_12_12_1$	$P6_322$
Cell dimensions		
a, b, c (Å)	57.4, 60.8, 120.0	70.7, 70.7, 48.5
α, β, γ (°)	90 90 90	90 90 120
Wavelength	0.9197	0.9197
Resolution (Å)	33.41 – 1.99 (2.02 – 1.99)	35.33 – 2.09 (2.13 – 2.09)
R_{merge}	0.056 (1.002)	0.036 (2.285)
$I / \sigma I$	12.7 (1.5)	29.7 (1.4)
CC (1/2)	1.00 (0.75)	1.00 (0.67)
Completeness (%)	99.6 (99.7)	99.0 (100)
Redundancy	5.7 (6.0)	12.1 (12.2)
**Refinement**		
Resolution (Å)	33.41 – 1.99 (2.06 – 1.99)	35.33 – 2.09 (2.17 – 2.09)
No. reflections	29339 (2891)	4512 (427)
R_{work} / R_{free}	0.189/ 0.227	0.259/ 0.288
No. atoms		
Macromolecules	2768	414
ligands	10	1
B-factors		
Macromolecules	77.08	57.24
ligands	70.51	53.42
Ramachandran favored (%)	99.17	0.00
Ramachandran allowed (%)	0.83	0.00
Ramachandran outliers (%)	0.00	0.00
Rotamer outliers (%)	0.00	0.00
R.m.s. deviations		
Bond lengths (Å)	0.013	0.009
Bond angles (°)	1.51	1.84

*Values in parentheses are for highest-resolution shell.

Table S2. Details of data collection and refinement statistics for the crystallographic data as deposited with the PDB.
